# Development and Preliminary Evaluation of a Conversational Agent Delivering Problem-Solving Therapy for Family Caregivers of Children With a Chronic Health Condition: Multiphase Mixed Methods Study

**DOI:** 10.2196/94175

**Published:** 2026-10-06

**Authors:** Weichao Yuwen, Liying Wang, Serena Jinchen Xie, Myra Divina, Xuehong Fan, William Kearns, Teresa M Ward

**Affiliations:** 1 School of Nursing & Healthcare Leadership, University of Washington Tacoma, 1900 Commerce Street, Campus Box 358421, Tacoma, WA, 98402, United States; 2 Florida State University, Institute on Digital Health and Innovation, College of Nursing, Florida State University,, Tallahassee, FL, United States, 1 (850) 644-3296; 3 University of Washington, Seattle, WA, United States

**Keywords:** health-wellbeing, family caregiver, usability study, mental health, conversational agent

## Abstract

**Background:**

Family caregivers of children with chronic health conditions experience substantial physical and mental health burdens, including burnout, anxiety, depression, fatigue, and sleep disturbances. Despite this need, validated digital mental health tools tailored to family caregivers remain limited. AI-powered conversational agents offer a promising approach for delivering on-demand, personalized mental health support, yet development and evaluation frameworks for this population are lacking.

**Objective:**

This paper describes the iterative development and formative evaluation of COCO (Caring of Caregivers Online), a conversational agent designed for family caregivers of children with chronic health conditions. COCO integrates problem-solving therapy (PST) and motivational interviewing (MI) within a human-in-the-loop development framework that progressed from rule-based interactions to a large language model (LLM)–powered conversational agent.

**Methods:**

COCO was developed across four phases: (1) caregiver persona and dialogue development based on PST and MI; (2) usability testing of a low-fidelity prototype with standardized patients in a single session of PST; (3) usability testing of a high-fidelity prototype with caregivers in a single session of PST (n=38); (4) integration of an LLM into COCO. The Wizard-of-Oz method was used across phases 2 and 3 to collect naturalistic dialogues and refine COCO’s conversational design. In phase 3, usability of COCO was assessed using the System Usability Scale (SUS). Caregiver emotions were measured before and after the session using 6 subscales of the PANAS-X. In phase 4, GPT-4 was integrated into COCO with few-shot learning and evaluated by research team members using the caregiver personas. Descriptive statistics were used to summarize quantitative measures. The MI principles and techniques used by COCO across the 4 phases were coded using the *Motivational Interviewing Treatment Integrity Coding Manual*.

**Results:**

In phase 1, 4 gold-standard dialogues were developed using caregiver personas. In phase 2, standardized patients described COCO as validating and identified its problem-solving and on-demand support as helpful for caregivers. In phase 3, COCO-Wizard-of-Oz achieved a mean SUS score of 75.6% (SD 12.9%), reflecting acceptable usability. Participants demonstrated significant improvement in negative affect, sadness, guilt, and fatigue following PST sessions (*P*<.05). In phase 4, an LLM-powered COCO was developed and demonstrated promising initial conversational capabilities. Across all phases, conversational quality showed progressively improved, with LLM-powered COCO achieving the highest density of MI techniques per turn (2.56) and greater balance across MI strategy types, particularly in seeking collaboration and reflection.

**Conclusions:**

COCO demonstrated feasibility and usability as a conversational agent for delivering protocolized therapeutic support to family caregivers of children with chronic conditions. The iterative, human-in-the-loop approach supported the development of empathetic and therapeutically grounded responses. More broadly, this study provides a structured framework for systematically integrating and refining evidence-based therapeutic approaches through iterative testing before LLM deployment.

## Introduction

In 2025, an estimated 63 million family caregivers provided unpaid care to adults and children living with chronic health conditions [1]. The roles and responsibilities of family caregivers often involve working full-time while attending to the needs (eg, medication administration, mobility, and attending health care provider appointments) of their loved one and their family’s needs, which often results in burnout and symptoms of anxiety, depression, sleep disturbances, fatigue, and distress [2-4]. Family caregivers need support and resources to assist with caregiving, specifically in managing stress [5]. These factors have contributed to a critical demand for effective ways to deliver self-management interventions that promote better self-care in family caregivers [6]. While various consumer-friendly informatics tools are available to patients and caregivers, barriers to access and lack of personalized design to meet the specific needs of caregiving populations have limited the technological adoption and efficacy of existing solutions. Similar to patients, family caregivers use technology for information and social support [7]. A recent systematic review reported on the lack of efficacy of existing health informatics tools for family caregivers, specifically the use of informatics tools to support family caregivers and their needs [8].

Studies have shown that digital mental health tools positively improve caregivers’ mental and physical health [9,10]. Caregivers shared that coping skills, emotion regulation, skill building, and education are important aspects of digital mental health tools [11]. Another study used a quantitative survey to measure pediatric blood marrow transplant caregivers’ activation after using the blood marrow transplant Roadmap IT tool. The results indicated that the caregiver’s anxiety has decreased, their quality of life has improved, and growth was found in activation compared to the baseline [12]. AI is one of the core technologies behind many smartphone apps, robotics, and medical technology. Leveraging AI can help rapidly scan data and provide tailored information based on individual needs. Although there are multiple positive impacts of health informatics tools found in several studies, validated AI-enhanced tools for family caregivers of children with chronic conditions are limited [8].

Conversational agents provide a promising opportunity for self-management support in health care. A conversational agent is a software program that leverages AI to simulate human-like conversations with the user through written text or spoken language [13,14]. The first health conversational agent dates back to 1966 with the introduction of ELIZA, which used memory and grammar rules to mimic a Rogerian and nondirective psychotherapist and constructed responses based on user input [15]. Since then, numerous studies have reported the development of conversational agents using language modeling techniques, and several systematic reviews have described the scope of the use of conversational agents in health care [16,17]. Recent advancements in language modeling, most notably in large language models (LLM), have opened new doors for AI technology to provide self-management support through natural language conversations. Generative AI models, such as the GPT series, Claude, and the open-sourced LLaMA, have set new standards for conversational agents, increasing user expectations and significantly improving the ability of these agents to provide support. For example, a recent systematic review reported the promising potential of mental health conversational agents showing strong efficacy in mitigating distress and depression symptoms [18]. In another study, researchers found that users can establish a therapeutic alliance with the conversational agent, which is a critical factor in successful therapy [19]. However, despite these potentials, there are risks in adopting AI-based conversational agents for mental health, including the lack of evidence-based data on accuracy, reliability, and interpretability [20-22]. The majority of AI-based systems have focused on delivering psychotherapy and psychoeducational content for the general population, and less attention is given to particular populations with specific needs and stress, such as family caregivers of children with chronic health conditions [18]. The National Alliance for Caregiving reported that family caregivers juggle several tasks simultaneously [23-25]. While conversational agents developed for the general population could offer much-needed mental health support, their focus is only a small fragment of the needs of family caregivers. Wang et al [26] explored the use of conversational agents to provide therapy for caregivers, identified both promises and limitations, and highlighted the need for more attention on developing conversational agents for caregivers and improving the quality of emotional response of these agents [26-28]. Recognizing the need, potential, and risk of conversational agents for family caregivers of children with a chronic health condition, the current study aimed to develop and evaluate a human-in-the-loop conversational agent designed to deliver problem-solving therapy (PST) for caregivers of children with chronic conditions specifically.

To provide services that offer targeted care and help relieve their physical and mental pressure, we developed Caring of Caregivers Online (COCO). COCO is a mobile application with an embedded chatbot providing family caregivers—particularly those with limited resources and high stress and burnout—with on-demand caregiving support and interactive self- and family-management skill development in English. The primary goal of COCO is to provide on-demand, personalized, and emotionally intelligent support and health solutions to reduce caregiver burnout, thereby promoting their health and well-being. Features and functions include evidence-based intervention components such as daily check-ins [29], weekly PST sessions [30], automated reminders for self-care [31], symptom self-tracking [24], on-demand health and caregiving question-and-answer capabilities [14], and tailored resource recommendations [32]. In this paper, we described the design, development, and preliminary testing of COCO’s key feature of delivering AI-enhanced PST to family caregivers.

## Methods

The development and iterative testing of COCO went through 4 phases outlined below. Phases 1 and 2 described the initial development of the rule-based conversational agent, phase 3 was the main usability study with prechat and postchat caregiver emotions evaluation, and phase 4 was an exploratory technical demonstration of an updated COCO using an LLM.

### Phase 1: Gold Standard Dialogue Development Informed by PST and Motivational Interviewing

#### Theoretical Foundation of COCO

COCO intervention was guided by PST to help caregivers reach self-management and adaptive coping of stressful life events associated with caregiving. PST is an evidence-based treatment approach that provides a framework for COCO to systematically assess the problem (ie, caregiving symptoms such as stress, worry, and sleep disturbances) at hand, set specific and realistic goals, and brainstorm solutions with users to generate adaptive coping strategies to resolve the problem and reach the goals [11]. The 7-step guideline of PST was used to develop the flow of the therapeutic dialogues underlying COCO (Textbox 1).

Textbox 1. **Problem-solving therapy (PST): 7-step guideline for Caring of Caregivers Online (COCO) dialogue.**

Step 1: Build a therapeutic relationship (eg, introduce COCO and the team behind COCO, and get to know the caregiver and their family).

Step 2: Explain the structure (eg, the PST process and short-term and long-term goals of the intervention).

Step 3: Identify and assess a caregiving symptom (eg, cause, frequency, context, and severity).

Step 4: Set goals to reduce the symptoms.

Step 5: Generate solutions to achieve the goals.

Step 6: Implement solutions.

Step 7: Evaluate solutions (eg, satisfaction, learning anything new about the symptom, and what can be done differently).

Additionally, we incorporated motivational interviewing (MI) principles into COCO to enhance the user’s willingness and readiness to change (Textbox 2). According to the stages of change model, individuals move through incremental processes as new behavior is cultivated, from not considering change to feeling ambivalent to getting prepared for change [33]. MI is both principle-oriented and technique-based, which together catalyze motivation enhancement and ambivalence resolution.

Textbox 2. **Motivational interviewing (MI) principles and techniques.**

**MI Principles**

- Cultivate change talk
- Soften sustained talk
- Strengthen partnerships
- Soften sustained talk
- Empathy

**MI techniques**

- Giving information
- Questioning
- Reflection (simple or complex)
- Affirmation
- Persuading with permission
- Seeking collaboration
- Emphasizing autonomy
- Normalizing (added by the authors)

#### Persona and Conversation Development

Persona-based prototyping is an established user-centered design method used to represent target users’ goals, behaviors, and needs in order to guide the iterative development of health technologies [34]. Personas serve as practical design tools that ground development decisions in the lived experiences of the intended population, helping ensure the technology is relevant, acceptable, and appropriately tailored before formal testing [35]. We developed 4 family caregiver personas through a systematic process integrating (1) a review of the empirical literature on caregiver burden and needs; (2) findings from our prior research with this population; and (3) participatory design sessions conducted with family caregivers of children with chronic health conditions and community-based experts working with these families [17,33,36-39]. Each persona was constructed to reflect common psychosocial symptom profiles documented in the literature, including worry, anxiety, fatigue, sleep deprivation, and tiredness, ensuring that COCO’s design addressed clinically and experientially relevant challenges faced by this caregiver population. Based on the personas developed, the clinicians (n=4) on the team (nurses and clinical psychologists) then created dialogues between COCO and caregiver personas, generating a total of 4 dialogues. In writing the dialogue, we incorporated the PST structure and MI principles, focusing on empathetic responses. Using the set of dialogues curated by the team, we created the low-fidelity prototype of COCO.

### Phase 2: Usability Testing of Low-Fidelity Prototype With Standardized Patients

#### Standard Patients

Standardized patients (SPs) are trained actors widely used in medical education and health technology research to simulate realistic clinical encounters in a controlled, reproducible manner [40]. We recruited 2 actors and trained them to role-play as family caregivers using the 4 personas developed in phase 1. Each SP was briefed on the psychosocial profile, symptom presentation, and caregiving context of their assigned persona prior to testing. This approach allowed for systematic and reproducible usability testing across personas while ensuring that interactions reflected clinically relevant caregiver experiences.

#### Wizard-of-Oz

The Wizard-of-Oz (WoZ) method is an established technique in conversational agent development that enables the collection of naturalistic user-system dialogues prior to full automation [41]. In WoZ testing, a user interacts with a mock interface believing it to be a functioning system, while a human “wizard” operating behind the scenes generates the system’s responses in real time. This approach allows researchers to evaluate interaction quality and refine dialogue design before committing to full technical implementation, making it particularly well-suited for early-stage conversational agent development [42]. For this study, 2 research team members with clinical experience served as COCO wizards, simulating responses that COCO was designed to deliver. The COCO-WoZ system architecture and technical setup have been described in detail in our previous publication [17]. SPs interactions with the mock interface were not informed of the wizard’s involvement, ensuring that their behavior and responses reflected naturalistic interaction with a conversational agent.

#### Usability Testing Procedure

Usability testing sessions were conducted via videoconferencing. Each of the 4 caregiver personas was tested in a 1-hour session, during which the SPs completed 2 PST sessions within that persona’s caregiving context. Following each session, usability data were collected through five open-ended questions addressing: (1) COCO-WoZ’s perceived personality, (2) system functionality, and (3) overall design quality.

### Phase 3: Usability Testing and Preliminary Effects of COCO-WoZ on Caregiver Positive and Negative Emotions

In this phase, we tested COCO-WoZ with family caregivers of children with chronic health conditions, examining system usability and changes in caregiver emotions in a pre-post study without a control.

#### Participants

We recruited participants using a snowball sampling method through flyers shared with online forums, clinics, and hospitals. Eligible participants must be over the age of 18 years, currently taking care of a child with a chronic condition, speak and understand English, have a computer or laptop, and have access to stable internet. Given the formative and exploratory nature of this study, no formal sample size or power calculation was performed. The study was designed to provide preliminary estimates of usability and short-term changes in affect and was not powered to demonstrate clinical effectiveness.

#### Procedure

We used the WoZ interface for COCO (COCO-WoZ) to deliver PST sessions. Participants reviewed study information, provided informed consent, and scheduled sessions with COCO-WoZ. Participants received an email reminder the day prior to their scheduled session. Before each session, the participants completed a brief survey rating their emotions at the moment. After completing the sessions, participants filled out a survey about their emotions and additional questions about their experience with COCO-WoZ.

#### Measures

We used the Positive and Negative Affect Schedule-Expanded Scale (PANAS-X) to measure changes in current emotions in users’ presessions and postsessions with COCO-WoZ [43]. We selected 6 subscales that included 34 items from PANAS-X to reduce survey burden. We selected 6 affective states: overall negative affect, specific negative affect (guilt, sadness, and fatigue), and specific positive affect (joviality and serenity). These were selected based on common caregiver symptoms and our hypothesis that these emotions would likely be affected by the therapy session. Each item was rated on a 5-point Likert scale of 1 (not at all) to 5 (very much). Each subscale consisted of 3 to 10 emotional items, respectively, and the subscale score was calculated by adding the individual item scores. Higher scores mean more intense emotions. User experience was measured using the System Usability Scale (SUS) [44]. The SUS is a 5-point Likert scale (1=“strongly agree” to 5= “strongly disagree”) with 10 items that assess system usability and learnability [45]. The total score of all items was converted to a 0‐100 scale, with a higher score suggesting better usability. A score of 70 or greater indicates acceptable usability [45].

### Phase 4: Development and Usability Testing of LLM-Powered COCO

Based on phase 3’s results, we continued improving COCO. In this final phase, we developed a GPT-4-based COCO delivering PST with integrated MI techniques and used persona-based methods to examine its preliminary performance. The details of the chatbot development process and testing are published in a separate manuscript [46].

#### Development

To develop GPT-4-based COCO, we used the emerging prompting methods at the time and created several versions of COCO and selected the best-performing model through human evaluation using a persona-based approach similar to phase 2. Details of the development and evaluation were reported elsewhere [46]. In the current study, we examined the dialogues generated from the best-performing model, which used few-shot learning with a brief description of COCO. The few-shot conversation examples were carefully crafted by our clinical team members using dialogues from phases 1‐3, each demonstrating one or more MI techniques to effectively engage users in behavior change while maintaining therapeutic alliance. The LLM-powered agent was deployed using Streamlit (Snowflake Inc), which enables users to interact with LLM-powered COCO through a web app.

#### Evaluation

Three clinicians in the team (2 nurses and 1 clinical psychologist) conversed with the LLM-powered COCO using caregiver personas and generated 12 dialogues for the current evaluation of coding MI techniques.

### Ethical Considerations

Phases 2 and 3 were reviewed by the University of Washington Institutional Review Board (IRB) and received exempt status (STUDY00007247). Phases 1 and 4 were not considered human subject research, and thus no IRB approval was needed. Participants eligible for the study provided informed consent before proceeding with study procedures.

### Data Analysis

Descriptive statistics were conducted to summarize quantitative measures in phase 3. We examined the distributions of the prechat and postchat PANAS-X 6 subscale scores, and paired-sample 2-tailed *t* tests were conducted to detect changes in emotions. To account for multiple comparisons across the 6 PANAS-X subscales, *P*-values were adjusted using the Holm procedure to control the family-wise error rate at *α*=.05. Effect sizes were calculated using Cohen *d* for paired samples, defined as the mean of the within-participant pre-post difference scores divided by the SD of the difference scores. Occasionally, a participant omitted 1 item in the PANAS-X. In this case, we did not compute the subscale score containing that emotion, and this participant was not included in the specific subscale *t* test.

We used the *Motivational Interviewing Treatment Integrity Coding Manual* developed by Moyers et al [23] to code MI principles and techniques reflected in participants’ dialogues with COCO across the 4 phases mentioned above (see Textbox 2 for MI techniques and principles). Initially, the research team members reviewed the phase 1 conversations and discussed coding. When a response did not fit an existing MITI category, a study-specific category was developed through discussion. For example, simple grounding was added to capture brief grounding or orienting responses (eg, “I see”) that were observed in the dialogue. The definitions and coding rules for these study-specific categories were discussed and agreed upon before the final coding was conducted. After that, one trained coder coded all COCO-generated responses. A second reviewer then examined the coding for accuracy and consistency. Any discrepancies were discussed between the coder and the reviewer until consensus was reached.

## Results

### Phase 1: Dialogue Development Informed by PST and MI

The conversation generally starts with COCO greeting the caregiver. After the caregiver generates a response, COCO will respond with an empathetic response such as “There seems to be a lot on your plate,” followed by a therapeutic response appropriate to the PST step in the conversation. The way the conversation is structured supports the design of COCO to be tailored to family caregivers (Textbox 3).

Textbox 3. **Example dialogues created in phase 1.**

*Example dialogue 1*

COCO: Earlier you mentioned that your worrying is causing you to feel on edge and anxious.

COCO: Is this correct?

Caregiver: Yes, that’s correct.

COCO: Okay.

COCO: You also mentioned that what is causing you to feel this way is worrying about Thomas.

COCO: Is this also correct?

Caregiver: Yep.

COCO: Is it safe to say that your worrying, the feeling of putting you on edge, and your anxiousness are all related?

*Example dialogue 2*

COCO: What would be a personal goal for you to address the sleep deprivation?

Caregiver: I want to get some sleep. But I can’t do that if I’m worried about my kid.

COCO: I understand.

COCO: It’s difficult to focus on taking care of yourself when your child is not well.

COCO: In the future, I'’d like us to explore the feeling of worry and work toward reducing that worry.

COCO: But for now, let’s talk about setting one goal to help you with your sleep deprivation.

### Phase 2: Usability Testing of Low-Fidelity Prototype With SPs

Participants overall found their experience affirmed by COCO-WoZ, felt supported, and perceived the chatbot as pleasant to interact with. Regarding COCO-WoZ’s perceived communication style, participants stated that the chatbot was helpful in providing actionable suggestions. They also liked the colloquial language the chatbot used, which was informal and conversational. Regarding current functions, participants expressed a preference for receiving solutions for their goals instead of being asked to generate their own, stating, “I want somebody to throw a couple [of] things, and we can together and figure out what sticks.” Additionally, participants noted that it was unclear whether to respond to the chatbot’s statement-like response (vs questions). For overall design quality, participants enjoyed the features of COCO-WoZ, such as providing vetted websites to offer information about online chatrooms to find emotional support or a community of caregivers. They particularly liked the flexibility of on-demand support that COCO-WoZ offered, stating, “It’s nice that it’s available. Anytime that would fit anybody’s schedule.”

### Phase 3: Usability Testing and Preliminary Effects of COCO-WoZ on Caregiver Positive and Negative Emotions

#### Participants

A total of 38 participants were enrolled in the study, with 6 participants having incomplete demographic data. Thirty-two participants were included in the analyses on usability testing and effects of COCO-WoZ on positive and negative emotions. The majority of the caregiver participants were female, above 36 years of age, with an annual income of US $100,000 or more. The majority of the children under care were under 10 years old and presented with a range of diagnoses, such as autism, arthritis, and diabetes (Table 1).

**Table 1.** Demographic characteristics of caregivers and children in the sample.

Characteristics	Participants, n (%)
Caregiver characteristics (n=32)	
Sex	
Male	6 (18.75)
Female	26 (81.25)
Age (y)	
26‐35	8 (25)
36‐45	18 (56.25)
46‐55	6 (18.75)
Income range (US $)	
Less than $19,999	2 (6.25)
$20,000-$39,999	1 (3.13)
$40,000-$59,999	2 (6.25)
$60,000-$79,999	3 (9.38)
$80,000-$99,999	2 (6.25)
≥$100,000	10 (31.25)
Do not know or want to share	12 (37.50)
Child characteristics (n=32)	
Sex	
Male	16 (50)
Female	16 (50)
Age (y)	
1‐5	11 (34.38)
6‐10	13 (40.63)
11‐15	6 (18.75)
>16	2 (6.25)
Diagnosis	
Anxiety	1 (3.13)
Arthritis	7 (21.88)
Asthma	2 (6.25)
Autism	8 (25)
Depression	1 (3.13)
Diabetes	3 (9.38)
Other	10 (31.25)

#### Usability Testing Feedback

Common feedback surrounded the response and conversation flow functionality, experience chatting with COCO-WoZ, and interface design. Some feedback from the participants’ initial session found the chatbot “helpful, but the response speed is quite slow” and “didn’t flow like a conversation.” Other participants also described their experience chatting with COCO-WoZ as a comforting and calming experience. One participant noted, “Using the bot, even now, was calming to me. It felt like it gave me some direction and hope.” Another shared, “Sharing my experience releases a lot of stress for me. Even just having someone listen.” We also gained feedback on improving the interface design. As one suggested, “I would appreciate it if there were some interesting photos embedded in the conversation.” The mean score for SUS was 75.6% (SD 12.9%). This gives COCO-WoZ a “B” level, implying that there are some problems with the chatbot that need to be addressed, even though the users were generally satisfied with the product [47]. The item-by-item analysis of the SUS matches the qualitative feedback: caregivers generally thought COCO-WoZ was easy to use, would like to use it, and felt confident in using it (average scores above 3 out of 5 for these items). Learnability was also high. One specific item that received a relatively lower usability rating was “I found the system very cumbersome to use,” which received an average of 2.21 out of 5. We thought this was mainly related to the slow response time in the WoZ system.

#### Changes in Positive and Negative Emotions

Compared to the presession, the participants showed significant improvements in the postsession of negative emotions with categories of negative affect, sadness, guilt, and fatigue (*P*<.05; Table 2).

**Table 2.** Results of negative and positive emotions.

Emotions	Mean (SD)	*t* test (*df*)	Holm-adjusted *P* value	Cohen *d*
Negative emotions				
Negative affect	−6.80 (6.59)	6.09 (34)	<.001	1.03
Guilt	−0.74 (1.09)	4.02 (34)	.001	0.68
Sadness	−2.53 (3.62)	3.95 (31)	.001	0.70
Fatigue	−1.94 (3.88)	2.92 (34)	.02	0.49
Positive emotions				
Joviality	+1.09 (6.24)	1.00 (32)	.05	0.17
Serenity	+1.21 (3.02)	−2.33 (33)	.33	0.40

### Summary of MI Techniques Across All Phases

The results show a progressive and varied use of MI techniques across the 4 phases.

In phase 1, a total of 4 sessions were coded for MI techniques, with a total of MI instances being 202. The top 3 MI techniques used were simple grounding (n=58, 28.71%), question (n=44, 21.78%), and reflection (n=30, 14.85%). Seeking collaboration and reflection also had significant usage, indicating a balance between engaging caregivers and ensuring empathetic responses (Table 3).

**Table 3.** Types of MI techniques used across each phase.

MI^a^ code	Phase 1	Phase 2	Phase 3	Phase 4
	MI count (n=202), n (%)	MI count (n=132), n (%)	MI count (n=2970), n (%)	MI count (n=385), n (%)
Seeking collaboration	25 (12.38)	21 (15.91)	396 (13.33)	80 (20.78)
Question	44 (21.78)	36 (27.27)	661 (22.26)	61 (15.84)
Reflection	30 (14.85)	22 (16.67)	266 (8.96)	58 (15.06)
Normalizing	5 (2.48)	3 (2.27)	167 (5.62)	53 (13.77)
Affirm	10 (4.95)	7 (5.3)	500 (16.84)	49 (12.73)
Persuade with permission	7 (3.47)	13 (9.85)	359 (12.09)	29 (7.53)
Giving information	21 (10.4)	10 (7.58)	167 (5.62)	28 (7.27)
Simple grounding	58 (28.71)	19 (14.39)	446 (15.02)	24 (6.23)
Emphasizing autonomy	2 (0.99)	1 (0.76)	8 (0.27)	3 (0.78)

a MI: motivational interviewing.

In phase 2, with 132 MI instances in 4 sessions, the use of questions increased to 27.27% (n=36), while seeking collaboration (n=21, 15.91%) and reflection (n=22, 16.67%) remained consistent. Interestingly, simple grounding decreased, reflecting a shift in conversational style as real-time interactions with SPs were tested (Table 3).

By phase 3, with a total of 2970 MI instances in 38 sessions, questions (n=661, 22.26%) and affirming (n=500, 16.84%) were among the most used MI strategies, showcasing an emphasis on helping caregivers clarify and reinforce positive behaviors. Seeking collaboration (n=396, 13.33%) and simple grounding (n=446, 15.02%) were also prominent, indicating COCO’s effectiveness in creating a collaborative environment (Table 3).

In phase 4, the LLM-powered version of COCO showed a more balanced distribution of MI techniques across its 385 instances in 12 sessions. Seeking collaboration (n=80, 20.78%) became the most frequently used technique, followed by questions (n=61, 15.84%) and reflection (n=58, 15.06%). This phase emphasized collaboration and empathetic understanding more than earlier phases (Table 3).

The average number of turns per session and MI techniques used per turn varied across phases. Phase 1 had 22.25 turns and 2.27 MI techniques per turn, while phase 3 had a similar 22.11 turns but a slightly higher MI-per-turn ratio (2.33), reflecting the robust integration of MI principles with caregivers. Phase 4 showcased COCO’s enhanced performance with the GPT model, which had fewer turns per session (12.55) but the highest MI-per-turn rate (2.56), illustrating its ability to deliver concentrated motivational support in fewer interactions (Table 4).

**Table 4.** The average number of motivational interviewing (MI) techniques per turn of conversations across 4 phases.

Phase	COCO^a^	Caregiver	Average number of turns	Average number of MI^b^	Average number of MI or turn
1	Team member	Persona-based team member	22.25	50.5	2.27
2	WoZ^c^-supported team member	Persona-based SP	25.75	33	1.28
3	WoZ-supported team member	Family caregiver	22.11	51.6	2.33
4	GPT^d^	Persona-based team member	12.55	32.08	2.56

a COCO: Caring of Caregivers Online.

b MI: motivational interviewing

c WoZ: Wizard-of-Oz.

d GPT: generative pre-trained transformer

## Discussion

This study aimed to develop and evaluate COCO, a conversational agent designed to deliver PST with MI techniques to family caregivers of children with chronic health conditions. The research unfolded in 4 distinct phases, each contributing to the refinement and evaluation of COCO. We started with laying foundational work for a theoretically sound intervention tailored to the unique needs of family caregivers. The subsequent phases used a mixed methods design and WoZ testing to assess the usability of COCO and its preliminary impact on user affective state in a prechat and postchat design. The last exploratory phase enhanced COCO’s performance using an LLM, using a similar number of MI techniques as humans, while exhibiting higher variability within the MI techniques used.

The evolution of COCO across the 4 phases demonstrates a clear progression in its ability to deliver PST with MI techniques, particularly as it transitioned from human-guided interactions to a fully LLM-powered system in phase 4. Initially, COCO relied heavily on foundational MI strategies, such as simple grounding and open-ended questioning, to engage caregivers. With further iterations of COCO, the integration of MI principles became more refined with increased emphasis on collaboration, reflection, and affirmation, creating a more balanced and empathetic dialogue. Using human-curated examples from the first 3 phases of the study, we developed an LLM-powered COCO. In the person-based testing environment in phase 4, this version of COCO was able to deliver an increased variety of MI techniques, fostering deeper engagement in fewer conversational turns. This shift indicates that as COCO’s conversational sophistication improved, it was able to more effectively respond to caregivers’ needs, providing a richer, more supportive environment. GPT-4’s ability to maintain consistency in MI use while enhancing collaboration and reflection highlights the potential of AI-driven agents to meaningfully replicate therapeutic techniques in a scalable and responsive manner.

The implementation of PST and MI through a chatbot platform like COCO presents unique opportunities and challenges. COCO offers on-demand support, potentially reaching caregivers with limited access to traditional therapy due to time constraints, geographic limitations, or financial barriers. However, personalizing COCO to capture the nuances of individual circumstances and emotional states is a significant hurdle, as is the challenge of establishing rapport—an essential aspect of PST and MI—through a chatbot interface [48,49]. Continued refinement of COCO’s conversational abilities and empathetic responses is needed to address these challenges. Ethical considerations also play a critical role, including ensuring user privacy and data security, and establishing appropriate escalation protocols for crisis situations.

We used the PANAX-S scale to measure changes in short-term affective state in phase 3. In the prechat and postchat design, caregivers showed significant reductions in negative emotions and an increase in the feeling of serenity. Although there is evidence showing the relationship between short-term affect change and long-term mental health, this study is only a first step toward examining the long-term therapeutic impacts of a conversational agent-delivered protocolized therapy [50,51]. In addition, we took the approach of WOZ testing in this phase, rather than a fully automated conversational agent. Due to the high-stakes nature of mental health support, we carefully chose this approach in our first pilot study with actual caregivers. The changes in emotions prechat and postchat were likely affected by a combination of the protocolized therapy delivered through COCO and the skilled human touches through the WoZ interface.

While the preliminary evaluation of the LLM-based conversational agent showed promise in adopting a range of MI techniques, challenges related to response quality and controllability remain. For example, we observed instances in which COCO repeated information shared by the caregiver rather than advancing the therapeutic conversation. This highlights the need to ensure that AI-supported interventions are not only responsive but also clinically appropriate, purposeful, and consistent with therapeutic goals [52]. Future work should prioritize clinical oversight and quality assurance throughout development, evaluation, and deployment. This may include expert-developed therapy guidance, structured review points to evaluate session quality, and mechanisms for clinicians to identify and correct problematic responses [53,54]. Technical approaches such as retrieval-augmented generation or multiagent systems [21,55] may support these goals, but their value should be evaluated in terms of whether they improve personalization, safety, and fidelity to PST and MI principles.

This study has limitations that should be considered. First, 3 out of the 4 phases of testing used persona-based approaches, limiting the variability of possible dialogues. Second, the initial COCO intervention was developed with a rule-based chatbot design, limiting its therapeutic scope. With the recent advancements in LLMs, we have started expanding COCO’s therapeutic scope while carefully examining its performance. Third, our analysis of MI techniques was exploratory in nature. The frequency of MI techniques and MI techniques per conversational turn were used as descriptive measures to compare conversational patterns across development phases and should not be interpreted as validated indicators of therapeutic quality or intervention effectiveness. Future studies could examine the relationship between MI technique use and clinically meaningful caregiver outcomes in chatbots. Finally, the pilot evaluation in phase 3 included a small convenience sample of predominantly well-educated, higher-income caregivers recruited from an urban university neighborhood, limiting the generalizability of the results. A larger definitive trial with a control condition and long-term clinical outcome evaluation is warranted.

In conclusion, COCO shows promise as an accessible approach for delivering PST and MI-informed support to family caregivers of children with chronic conditions. The human-in-the-loop development process helped refine COCO’s conversational design and improve the relevance and responsiveness of its interactions. The LLM-powered version of COCO exhibited a broader range of MI techniques and greater flexibility in applying these techniques during conversations. While these findings are encouraging, they are exploratory and do not establish therapeutic effectiveness. Further research with larger and more diverse caregiver populations is needed to evaluate COCO’s clinical impact, optimize personalization, and improve the controllability and safety of LLM-based interventions.

## Abbreviations

COCO: Caring of Caregivers Online
IRB: institutional review board
LLM: large language model
MI: motivational interviewing
PANAS-X: Positive and Negative Affect Schedule - Expanded Scale
PST: problem-solving therapy
SP: standardized patient
SUS: System Usability Scale
WoZ: Wizard-of-Oz
